# Machine learning prediction of progressive subclinical myocardial dysfunction in moderate aortic stenosis

**DOI:** 10.3389/fcvm.2023.1153814

**Published:** 2023-05-30

**Authors:** Mayooran Namasivayam, Thomas Meredith, David W. M. Muller, David A. Roy, Andrew K. Roy, Jason C. Kovacic, Christopher S. Hayward, Michael P. Feneley

**Affiliations:** ^1^ Department of Cardiology, St Vincent’s Hospital, Sydney, NSW, Australia; ^2^ Faculty of Medicine and Health, University of New South Wales, Sydney, NSW, Australia; ^3^ Heart Valve Disease and Artificial Intelligence Laboratory, Victor Chang Cardiac Research Institute, Sydney, NSW, Australia; ^4^ Vascular Biology Laboratory, Victor Chang Cardiac Research Institute, Sydney, NSW, Australia; ^5^ Icahn School of Medicine at Mount Sinai, Cardiovascular Research Institute, New York, NY, United States; ^6^ Cardiac Mechanics Laboratory, Victor Chang Cardiac Research Institute, Sydney, NSW, Australia

**Keywords:** aortic stenosis, echocadiography, machine learning, LV dysfunction, neural network

## Abstract

**Background:**

Moderate severity aortic stenosis (AS) is poorly understood, is associated with subclinical myocardial dysfunction, and can lead to adverse outcome rates that are comparable to severe AS. Factors associated with progressive myocardial dysfunction in moderate AS are not well described. Artificial neural networks (ANNs) can identify patterns, inform clinical risk, and identify features of importance in clinical datasets.

**Methods:**

We conducted ANN analyses on longitudinal echocardiographic data collected from 66 individuals with moderate AS who underwent serial echocardiography at our institution. Image phenotyping involved left ventricular global longitudinal strain (GLS) and valve stenosis severity (including energetics) analysis. ANNs were constructed using two multilayer perceptron models. The first model was developed to predict change in GLS from baseline echocardiography alone and the second to predict change in GLS using data from baseline and serial echocardiography. ANNs used a single hidden layer architecture and a 70%:30% training/testing split.

**Results:**

Over a median follow-up interval of 1.3 years, change in GLS (≤ or >median change) could be predicted with accuracy rates of 95% in training and 93% in testing using ANN with inputs from baseline echocardiogram data alone (AUC: 0.997). The four most important predictive baseline features (reported as normalized % importance relative to most important feature) were peak gradient (100%), energy loss (93%), GLS (80%), and DI < 0.25 (50%). When a further model was run including inputs from both baseline and serial echocardiography (AUC 0.844), the top four features of importance were change in dimensionless index between index and follow-up studies (100%), baseline peak gradient (79%), baseline energy loss (72%), and baseline GLS (63%).

**Conclusions:**

Artificial neural networks can predict progressive subclinical myocardial dysfunction with high accuracy in moderate AS and identify features of importance. Key features associated with classifying progression in subclinical myocardial dysfunction included peak gradient, dimensionless index, GLS, and hydraulic load (energy loss), suggesting that these features should be closely evaluated and monitored in AS.

## GRAPHICAL ABSTRACT

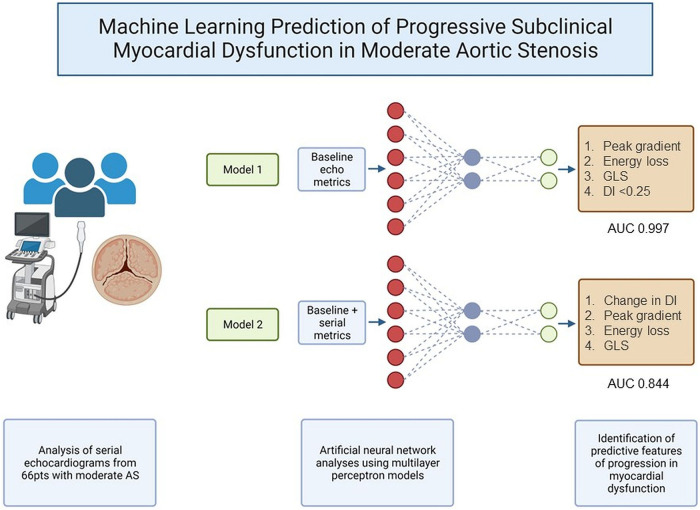

## Introduction

The understanding of aortic stenosis (AS) is rapidly evolving. “Moderate severity” AS is actually a complex and poorly understood entity (1–7). It involves hydraulic load on the left ventricle, leading to fibrosis and adverse clinical outcomes, with rates approaching those of severe AS (5). Moderate severity aortic stenosis is no longer considered a benign, early stage of disease, as was once thought (1–4). The importance of subclinical myocardial dysfunction in moderate AS has recently been highlighted (6). However, factors associated with the progression of subclinical myocardial dysfunction in moderate AS are not well understood. Mechanistically, myocardial dysfunction is related to hydraulic load, which, in turn, relates to valve stenosis severity (7). Metrics of valve stenosis severity are challenging to interpret even in truly severe AS, let alone in moderate AS when metrics can be more uncertain and discordant (8, 9). Machine learning, and specifically artificial neural networks (ANNs), can identify patterns in datasets to predict risk and identify features of importance. We sought to use machine learning to identify whether metrics of valve stenosis severity could accurately predict the progression of subclinical myocardial dysfunction in moderate AS, and we additionally sought to identify which valve stenosis metrics were most important to this progression. We believe that such analyses could provide insights into the basis for progressive deterioration and adverse outcome in what is traditionally considered “early stage” disease.

## Methods

### Subjects

We evaluated the St. Vincent's Hospital and Clinic Echocardiography databases to identify studies between 2016 and 2021 with moderate severity AS, as determined by a text-search of report conclusions [reports are finalized by an imaging cardiologist or a senior cardiology fellow (minimum PGY7)] and confirmation of mean gradient <40 mmHg. A total of 336 patients were identified with baseline imaging of adequate quality for strain analysis in apical 2, 3, and 4-chamber views, of whom 100 had serial imaging but only 66 had serial imaging of adequate quality for strain analysis at both index and follow-up.

### Echocardiographic analysis

A reanalysis of raw echocardiographic images was performed at the Heart Valve Disease and Artificial Intelligence Laboratory at the Victor Chang Cardiac Research Institute. Left ventricular global longitudinal strain (GLS) analysis was performed by using TomTec Arena software, with the apical 2, 3, and 4-chamber views (10, 11). Studies were included for analysis only if the left ventricular endocardium could be visualized and endocardial tracking was accurate in the apical 2, 3, and 4-chamber views throughout the cardiac cycle. Aortic valve stenosis severity was assessed using aortic valve area (AVA) (as determined by continuity equation), mean and peak transvalvular gradients, and dimensionless index (DI). Valvular hydraulic load was assessed using energy loss, modified from the method first described by Garcia et al. (12). Information on body surface area was not available in our database, and therefore, we evaluated total (rather than indexed) energy loss. As flow conditions are important for the metrics of aortic valve stenosis severity, transvalvular flow rate (Q) was also measured as the ratio of stroke volume to ejection time (13, 14).

### Neural network analysis

We utilized ANN analysis with multilayer perceptron models to identify features of valve stenosis severity from both baseline and serial echocardiography that could predict change in GLS between the index and the follow-up studies. We defined the target of models as change in GLS (classified by ≤ or > cohort median change). We constructed two models. The first used only inputs from baseline echocardiography. The second included additional inputs requiring data from serial echocardiography to account for the effects of dynamic change in metrics. We encoded inputs to optimize model efficiency, for example by binarizing key metrics at established diagnostic thresholds (such as AVA 1.0 cm^2^, DI at 0.25. Q ≤ 210 ml/s) (13, 15, 16). Inputs for the first model included the following: AVA ≤ 1.0 cm^2^, DI < 0.25, transvalvular flow rate ≤210 ml/s, peak gradient, mean gradient, left ventricular GLS, and energy loss. Inputs for the second model included model 1 inputs and additionally included the following: time between studies, change in AVA between studies, change in mean gradient between studies, change in dimensionless index between studies, change in energy loss between studies, and change in flow rate between studies. Both models used a single hidden layer architecture and a 70%:30% training/testing structure. This meant that models were trained on 70% of the cohort that was randomly selected and tested on the remaining unseen 30% of the cohort for validation, in order to avoid overfitting (17).

Model performance was assessed using prediction accuracy (% correct predictions), area under the receiver operating curve (AUC), and gain and lift functions. Features of importance were assessed using normalized importance scoring, with the most important feature scoring 100%, and other features were scaled accordingly as a proportion of importance.

Analyses were conducted using IBM SPSS Version 26.0 (IBM, Armonk, NY).

## Results

After exclusion criteria were implemented, 66 subjects (44 males, 22 females), aged 79 ± 10 years, with serial, strain-quality imaging over a median follow-up interval of 1.3 years (IQR: 0.8–2.0 years), remained. Baseline AS severity metrics are outlined in Table 1. Key valve severity metric changes from baseline to follow-up are noted in Table 2. All metrics of severity trended worse over time, with statistical significance reached by peak gradient, mean gradient, and peak velocity. GLS worsened over follow-up (baseline mean GLS −16.7%, mean change in GLS +0.74%, median change +0.48%), although the change in GLS was of borderline significance (*p* = 0.06, paired t-test).

**Table 1:** Baseline characteristics.^a^

Age (years)	79 ± 10
Male/female	44 (66%)/22 (33%)
Peak gradient (mmHg)	39 (33–44)
Mean gradient (mmHg)	23 (20–27)
Aortic valve area (cm^2^)	1.1 (0.8–1.5)
Dimensionless index	0.34 (0.26–0.40)
Transvalvular flow rate (ml/s)	256 (199–297)
Energy loss (cm^2^)	1.2 (0.8–1.5)
Global longitudinal strain (%)	−16.7 ± 4.3
Left ventricular ejection fraction (%)	61 ± 8
Left atrial dimension (mm, parasternal long axis)	42 ± 7
Interventricular septal thickness (mm)	11 (10–13)
Posterior wall thickness (mm)	11 (10–12)
Estimated pulmonary artery systolic pressure (mmHg)	27 (23–34)

^a^ Data presented as mean ± SD if normally distributed based on the skewness statistic between −0.5 and +0.5, aside from age and LVEF which, while not normally distributed, are reported here as mean ± SD per convention. Non-normal data presented as median (IQR).

**Table 2:** Changes in the metrics of AS severity at follow-up.^a^

Metric of severity	Follow-up	*p*-Value^b^
AVA (cm^2^)	1.00 (0.77–1.26)	0.057
Peak gradient (mmHg)	43 (34–49)	0.02
Mean gradient (mmHg)	25.1 (21.0–29.9)	0.031
Peak velocity (m/s)	3.3 (2.9–3.5)	0.02
Dimensionless index	0.30 (0.23–0.36)	0.12

^a^ Results are median (IQR).

^b^ As compared with baseline.

**Figure 1:**
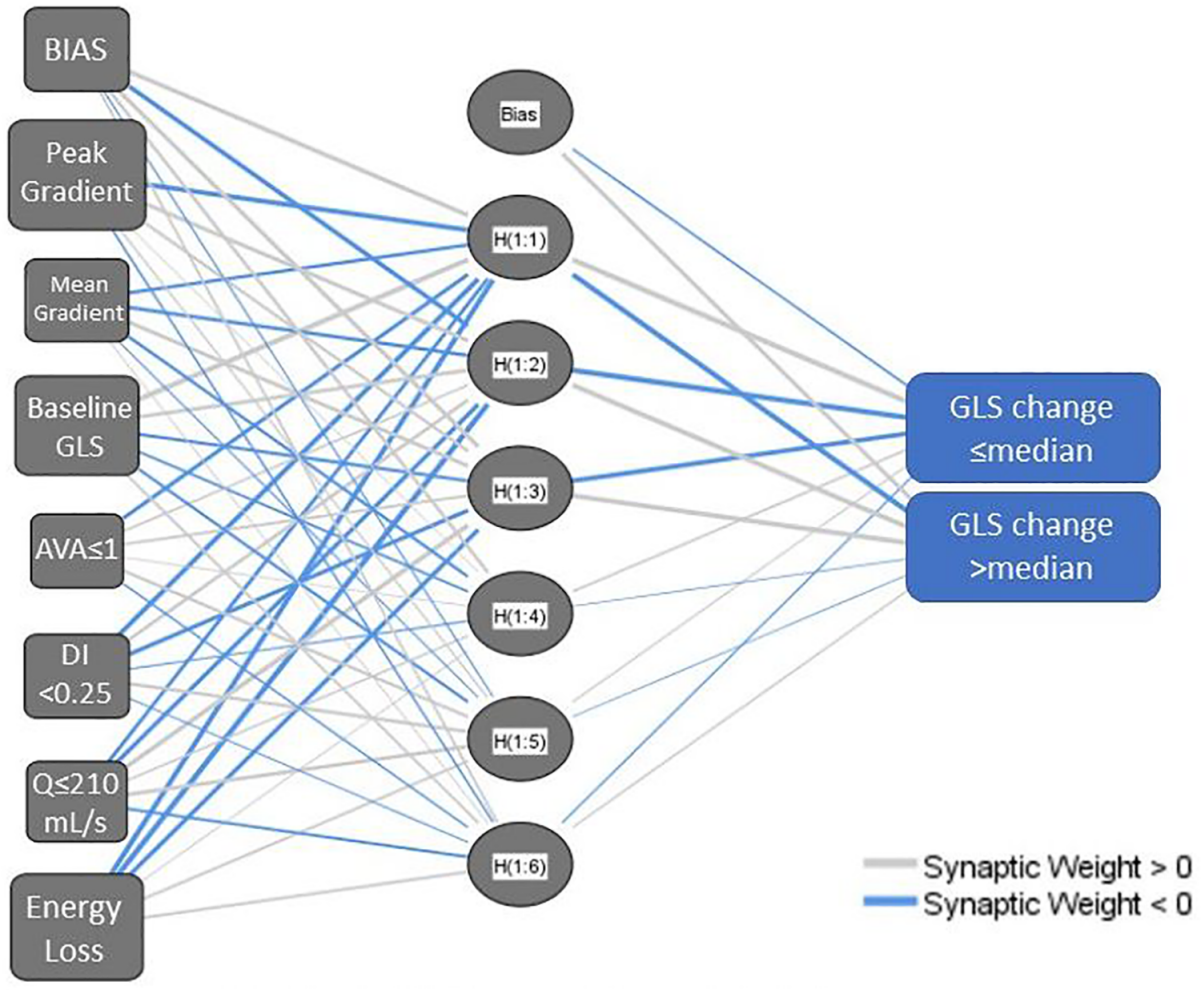
Network diagram for a multilayer perceptron neural network model using baseline aortic stenosis severity metrics (model 1). AVA, aortic valve area; DI, dimensionless index; GLS, left ventricular global longitudinal strain; Q, transvalvular flow rate. The network structure includes a bias node at the input layer and hidden layer and has multiple nodes in the hidden layer, hence H(1:1), H(1:2) etc.

As described, multilayer perceptron models were created initially from the metrics of AS severity from baseline echocardiogram alone (Model 1), and subsequently, incorporating parameters accounting for dynamic change in variables between the index and the follow-up studies (Model 2). The inputs and architecture for Model 1 are shown in Figure 1.

**Figure 2:**
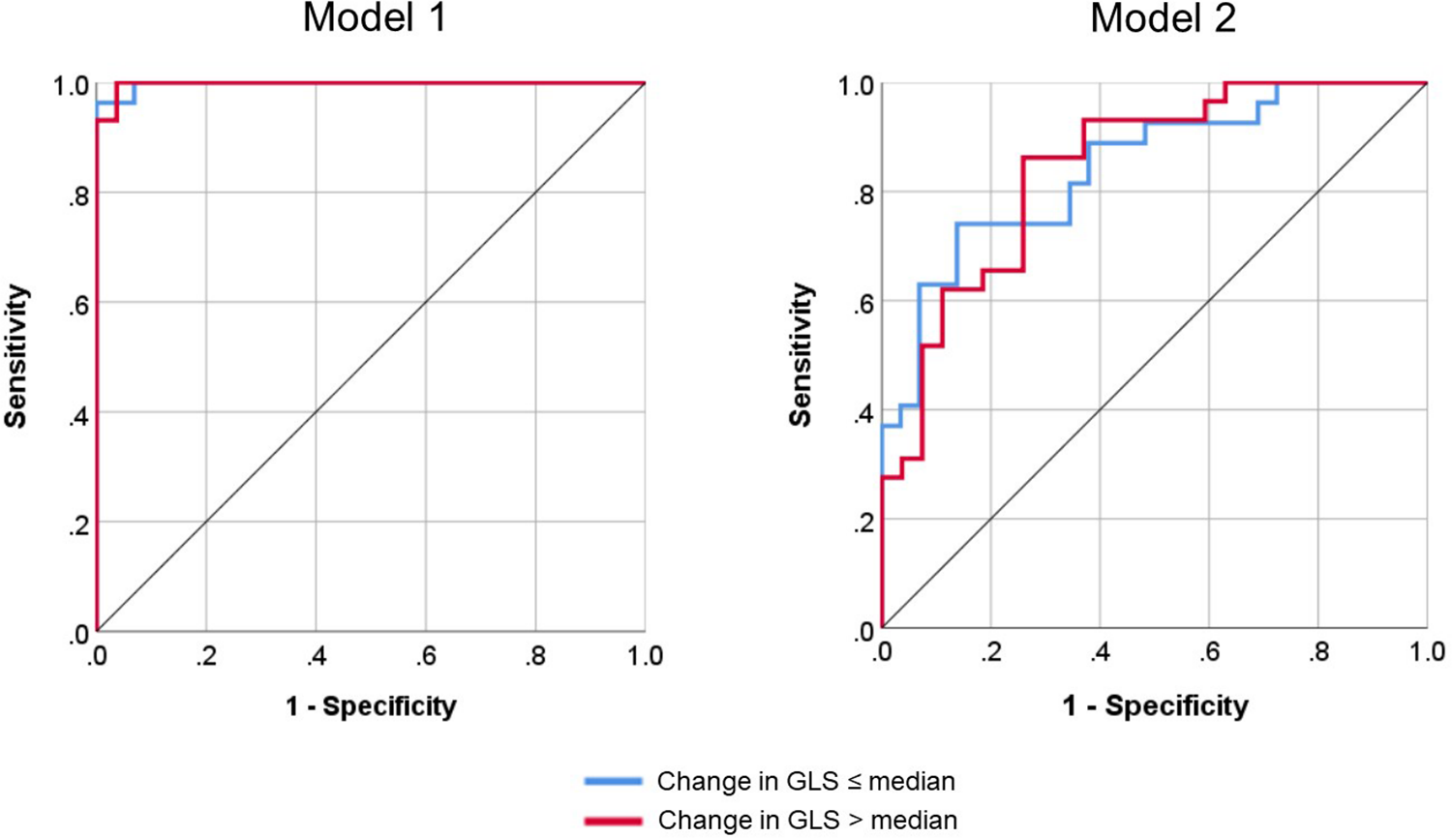
Receiver operating curves for Model 1 (baseline metrics only) and Model 2 (baseline metrics and dynamic change) for the classification of global longitudinal strain (GLS) progression.

**Figure 3:**
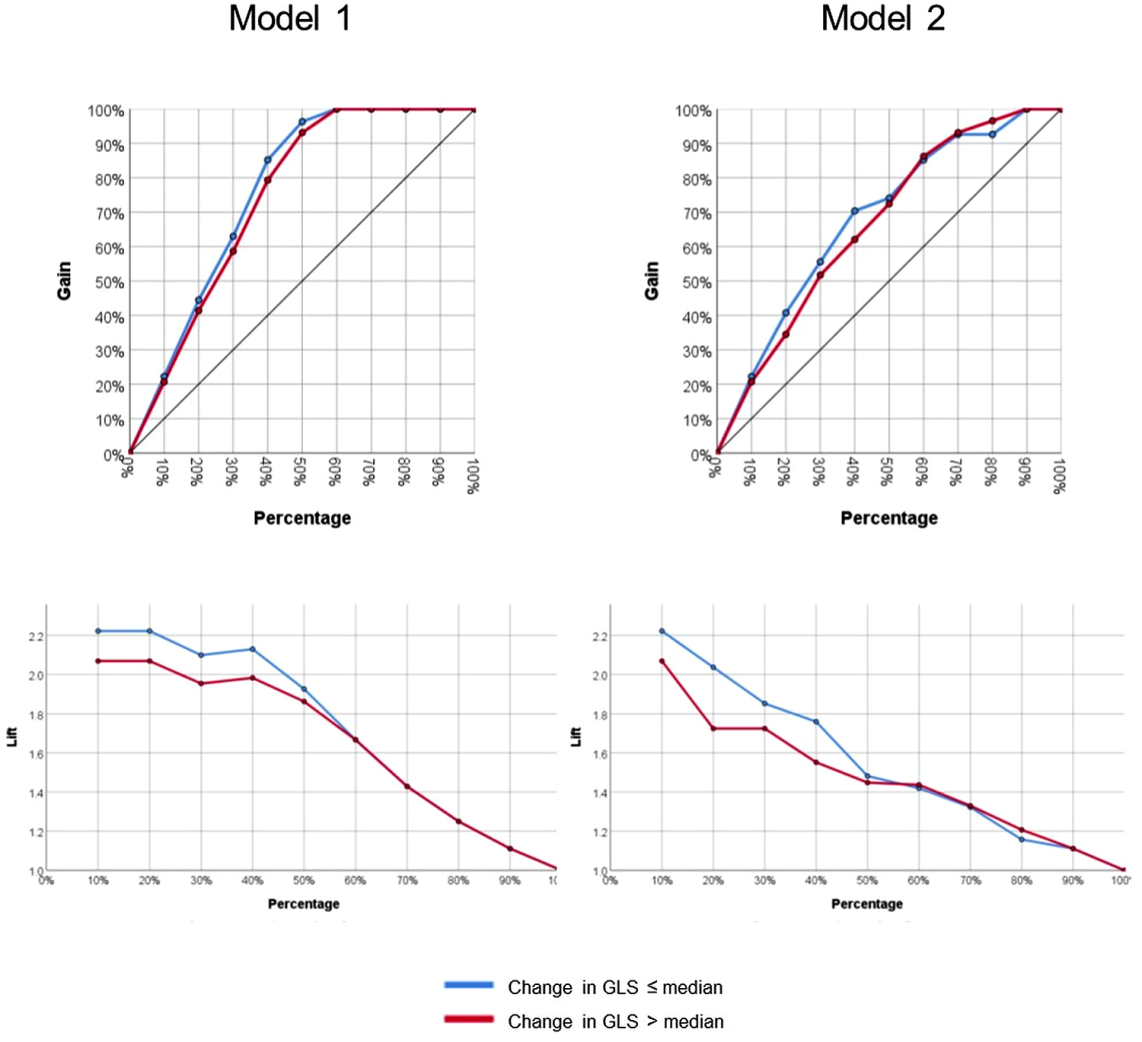
Gain and lift functions for Model 1 (baseline metrics only) and Model 2 (baseline metrics and dynamic change).

**Figure 4:**
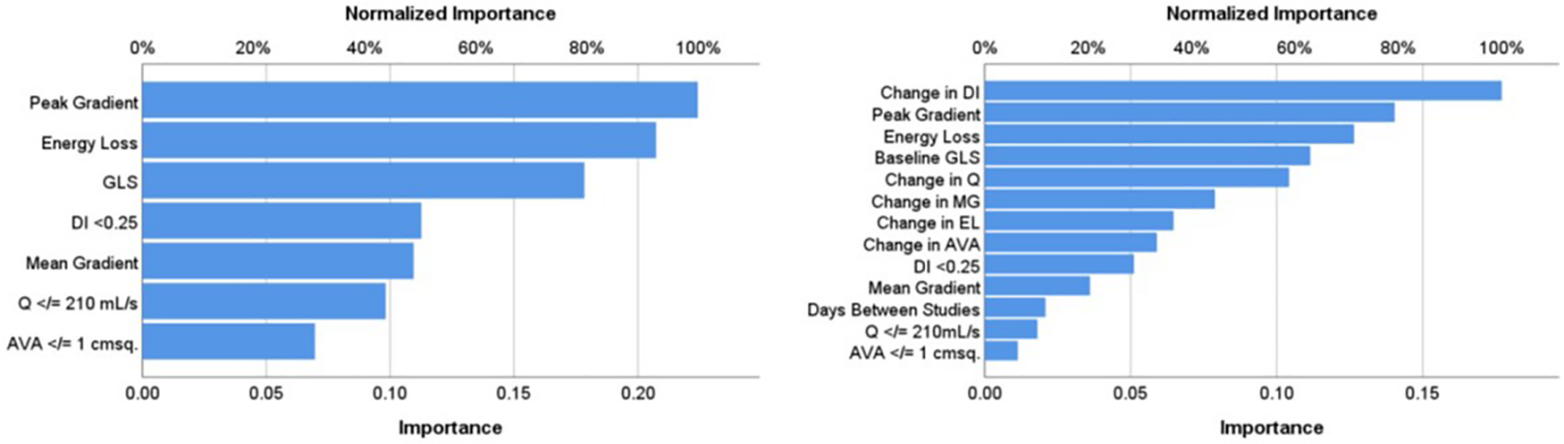
Relative importance of input features in Model 1 (baseline metrics) and Model 2 (baseline metrics and dynamic change). AA, aortic area at sinuses of Valsalva; AVA, aortic valve area (in cm^2^); DI, dimensionless index; EL, energy loss; GLS, left ventricular global longitudinal strain; MG, mean gradient; Q, transvalvular flow rate.

Model 1 yielded a prediction accuracy rate of 95% in training and 93% in testing, with an AUC of 0.997, while Model 2 yielded a prediction accuracy rate of 84% in training and 68% in testing, with an AUC of 0.844. Receiver operating curves for both models are shown in Figure 2. Gain and lift functions are shown in Figure 3. The relative feature importance in each model is shown in Figure 4. In Model 1, the four most important predictive baseline features (reported as normalized % importance relative to the most important feature) were peak gradient (100%), energy loss (93%), GLS (80%), and DI < 0.25 (50%). In Model 2, the top four features of importance were change in dimensionless index (100%), baseline peak gradient (79%), baseline energy loss (72%), and baseline GLS (63%).

## Discussion

Studies have shown that patients with moderate AS have poor overall survival (1–5) that is comparable to patients with severe AS (5), suggesting that current classification systems are inaccurate and understanding of the disease and its progression are limited. Moreover, studies have shown that the subclinical myocardial dysfunction in AS is associated with adverse clinical prognosis (6). AS quantification is challenging, even when disease is severe, but is particularly challenging in earlier, moderate stages of disease. Knowing which features are most important can inform echocardiographic assessment, clinical decision-making, and overall patient management. In this study of moderate AS, we have shown that progression in subclinical myocardial dysfunction can be accurately predicted, using neural networks, by simple echocardiographic metrics of AS severity. Moreover, we have shown that some features of AS severity are considerably more important than others in predicting change in myocardial function. The most important features are peak gradient, baseline GLS, DI, and energy loss.

These findings are important because they highlight that aortic valve stenosis severity is a major contributing factor to the load faced by the left ventricular myocardium, even in moderate stages of the disease. Moreover, this load and subsequent myocardial dysfunction can progress with time, even over a relatively short follow-up interval (median time 1.3 years). This is important because it means that ongoing exposure to load, even at moderate AS severity, is likely to have a continued and progressive adverse impact upon myocardial function, which may be irreversible (18, 19).

Our findings also highlight which key parameters are important to progression in myocardial dysfunction in moderate AS. The importance of DI in both our models affirms recent literature demonstrating the value of this metric (20). Of note, AVA did not rank highly in importance in either of our models. This finding of superiority of DI to AVA in our models, most likely reflects the flow dependency of AVA and relative flow independency of DI (13). As expected, baseline GLS was an important feature relevant to the degree of GLS progression. This finding supports the increasing calls to better quantify myocardial dysfunction using tissue deformation in AS, both in moderate and in severe disease (6, 21). Early detection of myocardial dysfunction could identify those at high risk of progression. Finally, energy loss, a marker of valvular hydraulic load, featured as important in our analysis. This metric is not routinely used in clinical practice but has a strong research foundation and has been shown to identify clinical risk in previous studies (12, 22, 23). Energy loss accounts for the changes in the composition of total energy (comprised of kinetic and static pressure energy that interchange, as well as gravitational potential energy that remains stable) as blood traverses a stenotic aortic valve. Energy loss accounts for the phenomenon of pressure recovery that occurs in the proximal aorta, whereby static pressure energy is “recovered” as blood enters the proximal aorta and kinetic energy reduces. Incomplete pressure recovery contributes to hydraulic load, and the degree of this recovery, and hence energy loss, is itself dependent on the ratio of effective aortic valve orifice area and aortic area. Conceptually, it is the best physiologic marker of hydraulic load faced by the ventricle as a result of aortic valve stenosis (12, 24), and its importance in contributing to deteriorating strain affirms that it adds clinical value. Our findings support increasing the role of energy loss quantification, which is easily measured from standard echocardiography, in assessment of AS. A particularly interesting finding was that peak gradient outperformed mean gradient as an input feature. Traditionally, mean gradient has served as the more frequently evaluated metric in AS; however, the spread of data in peak gradient (IQR: range 11 mmHg vs. 7 mmHg for peak vs. mean gradient) likely allowed a greater resolution to detect differences in moderate stages of the disease, where gradients are lower. This finding has potential implications for low-gradient severe AS, where the focus on mean gradient perhaps should be shifted to peak gradient.

Another important finding in our study was the demonstration of the power of machine learning in the assessment of echocardiographic data (25–30). We have shown that prediction models with high accuracy and explainability (i.e., the ability to explain the basis of model formulation and identify which features are of particular importance in generating the model) can be developed using easily acquired echocardiographic metrics. Machine learning approaches can identify nonlinear and interaction patterns more ably than conventional statistical modeling, allowing these approaches to provide a greater insight into the understanding of disease processes and progression from existing clinical data repositories. Our findings support other data, which has strengthened the calls to improve diagnosis and phenotyping of AS through novel machine learning approaches (31–34).

While our findings are important and add to the body of literature supporting the need for greater advanced image phenotyping and risk prediction in moderate AS, some limitations should be discussed. First, our study is a single center, retrospective observation of prospectively collected, longitudinal data. To mitigate the limitation of a single-center approach, we used sample splitting to create an independent training and testing set, to avoid overfitting models to the trained dataset (17). It is important to note that the limitations of a single center machine learning approach are less relevant to our study because the goal of this study was not to create a broadly usable risk calculator. Rather, the motivation of this work was to (1) demonstrate that baseline echocardiographic metrics of AS severity can be used to predict change in myocardial function in moderate AS and (2) identify which parameters were most important to this change. In this sense, external validation is less important, as the study was intended to disclose key concepts in the understanding of disease progression in moderate AS, rather than create an externally usable model that would benefit from an external validation of generalizability. Having said this, the general inherent biases of a single-center study and reliance on subjects with a single-center follow-up must be acknowledged. With regard to the comparatively small sample size, we used a cohort that is well phenotyped and individually reanalyzed serial images with strict quality control. We took this approach in contrast to using a large-sized cohort with a shallow phenotyping approach (e.g., automated data repository metric extraction), which while important in its own right, can introduce noisy, missing, and/or inaccurate data in an initial survey. Our work demonstrates an early experience with this approach, which has yielded valuable insights, and we encourage larger studies using database mining that could validate our findings. Our follow-up time was relatively short (median 1.3 years). This partly explains the modest changes in GLS over the study period. Indeed, our observed GLS change over the study period falls within the range of inter- and intraobserver variability reported elsewhere for GLS (35). Nevertheless, we demonstrated a clear trend in worsening GLS, and it is unlikely that random distribution of error would obscure the overall trend. Hence, despite this relatively short time interval, we were able to show that baseline AS metrics could accurately predict change in myocardial function over time, suggesting that the results would only have been amplified with a longer follow-up. Prospective studies with longer follow-up times could confirm and extend the findings of this study.

## Conclusions

Moderate AS is a high-risk clinical entity, and subclinical myocardial dysfunction is important for this risk. Factors important to the progression of myocardial dysfunction in moderate AS are not well known. In this study, we have shown that neural network analysis could accurately predict change in subclinical myocardial dysfunction in moderate AS and identify which features were most important to this change. The key features included peak gradient, DI, baseline GLS, and energy loss, suggesting that these parameters should be evaluated carefully while assessing patients with moderate AS.

## Data Availability

The datasets presented in this article are not readily available because IRB restrictions prohibit sharing of data beyond our institution but can be considered upon reasonable request. Requests to access the datasets should be directed to mayooran.namasivayam@unsw.edu.au
